# Editorial: Computational approaches to build therapeutic paradigms targeting genes, proteins and pathways against neglected tropical diseases (NTDs)

**DOI:** 10.3389/fgene.2023.1183034

**Published:** 2023-05-05

**Authors:** Anupam Nath Jha

**Affiliations:** Computational Biophysics Laboratory, Department of Molecular Biology and Biotechnology, Tezpur University, Tezpur, India

**Keywords:** tropical diseases, leishmaniasis, leprosy, neglected tropical diseases, protozoan parasites

Neglected tropical diseases (NTDs) are a set of parasitic and bacterial infections that predominantly affect populations in developing countries. These diseases often lack effective treatments, and developing new therapies can be challenging due to limited funding, research infrastructure, and access to patient populations. Computational approaches have emerged as a promising approach for identifying new therapeutic targets and designing novel lead molecules for NTDs. One key part of focus for computational methods in NTDs is the identification of genes, proteins, and pathways that are indispensable for the survival and proliferation of the causative pathogens (Bora and Nath Jha, 2019; Bora and Jha, 2020). This can be realized through a range of techniques, including genome-wide association studies, network analysis, and machine learning algorithms. Once these targets are identified, researchers can use computational methods to design and optimize lead candidates that explicitly target these pathways. However, there are numerous limitations to computational approaches for developing therapies for NTDs. One key challenge is the dearth of reliable data on the molecular mechanisms underlying these diseases, which can limit the accuracy and effectiveness of computational models. Additionally, there are often substantial differences in the genetic makeup and clinical presentation of patients with NTDs, which can make it challenging to develop therapies that are effective across diverse populations. Despite these constraints, computational approaches have noteworthy prospectives to advance our understanding of NTDs and develop new therapies. By leveraging cutting-edge technologies such as next-generation sequencing, artificial intelligence and machine learning, investigators can analyze massive expanse of data and produce novel insights into the underlying biology of NTDs (Kumari et al*.*, 2023). Ultimately, these insights can be utilized to design further effective and targeted therapies that can aid to alleviate the burden of NTDs on vulnerable populations. This Research Topic comprises four articles on HLA mapping in leprosy, signaling mechanisms in unicellular protozoans, divisome components in bacterial NTD and system level insights into interleukin (IL) regulation in leishmania.

In their study, Li et al. investigated the development and variation of leprosy-associated HLA alleles (human leukocyte antigen) in Han and minority populations in southern China. Leprosy is a chronic infectious disease that affects the skin, peripheral nerves, and mucous membranes. The study found that certain genes, specifically those located on chromosome 6p21 and HLA-DQB1 gene were significantly associated with an increased risk of leprosy among Han and minority populations in southern China. The study also highlights how genome-wide association studies have identified single nucleotide polymorphisms (SNPs) within the MHC region as the most prominent inherited variant connected with leprosy. The MHC region is responsible for coding HLA genes that regulate the immune response. The article acknowledges the challenges associated with associating HLA genes with susceptibility to leprosy. The genetic factors associated with leprosy susceptibility differed between ethnic groups, indicating a complex genetic basis. The study suggests that HLA genotyping could be used to identify individuals at increased risk of leprosy and to develop personalized treatment strategies. rs75324027 locus located between non-HLA-DRB1 and HLA-DQA1 intergenomic region and chr6:32626438-A-T in the HLA-DQB1 gene in M-S (Han leprosy patients in south China) and M-SM (Leprosy patients of ethnic minorities in south China) disease were found to be the most significant. The diversity of HLA alleles within populations and the complex nature of the disease contribute to the complexity of the study. The research significantly increases knowledge of disease risk factors and suggests an association of new biological pathways with leprosy.

All eukaryotes share the Cdc42 and Rac GTPase-dependent signaling pathways; however lower eukaryotes like protozoan parasites lack these annotations or characterizations (Hodge and Ridley, 2016). The Rho family proteins are regulatory molecules for actin cytoskeleton dynamics, with Cdc42, Rac1, and RhoA being subfamilies. Several studies have characterized Cdc42, Rac1, and RhoA members of the Rho family in humans. PAKs and actin assembly protein families are conventionally found during evolution, while Ser/Thr and cytosolic Tyrosine kinase, adaptor family proteins present in humans are non-conventional. Umarao et al., endeavored to identify and investigate the potential of CRIB-containing effector protein as a drug target in lower eukaryotes, particularly in disease-causing protozoa. They identified a number of CRIB-containing effector proteins in *Acanthamoeba castellani*, *Dictyostelium discoideum*, *Entamoeba histolytica*, *Giardia lamblia* and *Trypanosoma cruzi*. For *Leshmania donovani*, a protein, Q4QEZO Ser/Thr Kinase family effector protein containing PAK catalytic domain, was reported but with no CRIB domain. And another E9BGF4 coronin homolog protein was also reported but its association with CRIB is yet to be explored. They concluded their investigation with the need to validate the proteins experimentally as a potential drug target.

The FtsQBL is a protein complex essential for cytokinesis, and its components are promising drug targets due to their essentiality. The conserved protein FtsQBL complex plays a crucial role in bacterial cell division in Neglected Tropical Diseases (NTDs). The NTDs are a set of bacterial infections that affect more than a billion people worldwide and are often resistant to existing therapeutics (Hotez et al*.*, 2014). Kaur and Lynn described the identification and mapping of the FtsQBL divisome components in bacterial NTDs (Cholera, Buruli ulcer, Leprosy, and Trachoma). The study utilized computational tools to identify and map the FtsQBL divisome components in various NTD pathogens and the researchers identified several potential drug targets within the FtsQBL complex, including FtsL, FtsQ, and FtsB. The authors have utilized hidden Markov models, profile-profile comparison, and structural modeling to identify the function of these homologs. The study highlights the importance of targeting conserved components of the bacterial cell division machinery as a strategy for developing new therapeutics against NTD pathogens.

Leishmaniasis is a disease caused by the Leishmania spp that resides in mammalian hosts. It is endemic in many countries, including India, which has been heavily affected (Saha and Jha, 2023). Cytokines play a role in the development of a Leishmania infection. Cytokine reciprocity can play a role in the modulation of the immune response during Leishmaniasis. Different cytokines have different effects on the immune response, and transcription factors such as ICSBP, IRF1, NFAT5 and C/EBP can play a role in this process. Computational modeling of the reciprocal regulation of IL12 and IL10 in Leishmaniasis can help one in understanding the signaling mechanism responsible for parasite survival. Khandibharad and Singh, describes the use of computational approaches to study the reciprocal regulation of the cytokines IL10 and IL12 in Leishmaniasis. IL10 and IL12 play important roles in the immune response to Leishmania infection, and their reciprocal regulation is critical for the control of the disease (Ma et al*.*, 2015). Khandibharad and Singh utilized a computational model to simulate the dynamic interactions between IL10, IL12, and other immune factors during Leishmania infection. The model predicted that IL10 production is regulated by a negative feedback loop involving IL12, whereas IL12 production is regulated by a positive feedback loop involving IL10 and other factors. NFAT5 is important for promoting IL12 synthesis and inhibiting IL10, while SHP-1 is important for dephosphorylating NFAT5 and regulating NFAT5 activity. NFAT5 can be considered as a point of intercession to scrutinize the parasite clearance regime. The study identified plausible drug targets for modulating the reciprocal regulation of IL10 and IL12 in Leishmaniasis, which could lead to the development of new therapies for the disease.

The Research Topic of works presented in this section sheds light on various aspects of NTDs, which are a set of bacterial and parasitic infections. The Research Topic of works highlighted in the article has advanced the research in the field of Neglected Tropical Diseases (NTDs) by providing insights into various aspects of these diseases. The studies have focused on diverse areas including epidemiology, transmission dynamics, control strategies, and the development of new drugs and diagnostic tools. In overall, the breadth of the papers in this Research Topic reflects significant progress in comprehending the essential omics knowledge for NTDs causing species, discovering potential therapeutically important targets, and utilization of sequencing datasets to decipher underlying and unexplored biological pathways involved in NTDs.
